# Planned missing data in early literacy interventions: A replication study with an additional gold standard

**DOI:** 10.1371/journal.pone.0249175

**Published:** 2021-03-29

**Authors:** Ralph C. A. Rippe, Inge Merkelbach

**Affiliations:** 1 Research Methods and Statistics, Institute of Education and Child Studies, Leiden University, Leiden, The Netherlands; 2 Learning and Behavior Problems in Education, Institute of Education and Child Studies, Leiden University, Leiden, The Netherlands; Iwate Medical University, JAPAN

## Abstract

**Introduction:**

In a digital early literacy intervention RCT, children born late preterm fell behind peers when in a control condition, but outperformed them when assigned to the intervention. Results did however not replicate previous findings. Replication is often complicated by resource quality. Gold Standard measures are generally time-intensive and costly, while they closely align with, and are more sensitive to changes in, early literacy and language performance. A planned missing data approach, leaving these gold standard measures incomplete, might aid in addressing the origin(s) of non-replication.

**Methods:**

Participants after consent were 695 p Dutch primary school pupils of normal and late preterm birth. The high-quality measures, in additional to simpler but complete measures, were intentionally administered to a random subsample of children. Five definitions of gold standard alignment were evaluated.

**Results:**

Two out of five gold standard levels improved precision compared to the original results. The lowest gold standard level did not lead to improvement: precision was actually diminished. In two gold standard definitions, an alphabetical factor and a writing-only factor the model estimates were comparable to the original results. Only the most precise definition of the gold standard level replicated the original results.

**Conclusion:**

Gold standard measures could only be used to improve model efficiency in RCT-designs under sufficiently high convergent validity.

## Introduction

Developing and validating effective interventions is a central goal of educational research. Experimental designs (random-controlled trials; RCTs) are currently the most powerful designs for testing the effectiveness of interventions. However, in educational settings they may be difficult to realize. Factors such as small sample sizes, the use of outcome measures with suboptimal validity [1] and indirect enforcement of the experimental intervention (via teachers) can compromise the experimental outcome. To examine the influence of aforementioned factors on the results of a study, the common approach is to replicate the study with a new, larger, sample, and with higher-quality measures. In practice, however, such replications are time and resource intensive by their very nature as they require recruitment and data collection from a new sample. An alternative approach is to use a planned missing data approach and administer high quality, time-intensive measures to a randomly selected subset of participants in the original study. In what follows we elaborate on the above concepts using an educational replication study targeting a group of susceptible preschool pupils (i.e. children born late preterm or small for gestational age) as an example.

### Differential susceptibility

Over the past decade, the differential susceptibility model ([2, 3]) has become universally applied in behavioral sciences. Central to this model is the notion that individuals carrying certain genetic or neurobiological markers may be more susceptible to the quality of their environment (e.g. various types of interventions), both for better and for worse. In contrast to the common diathesis stress model [4], which postulates that subgroups with certain biological vulnerabilities will fall behind when conditions are adverse, the differential susceptibility postulates that subgroups will fall behind when conditions are adverse, but will succeed–and even surpass less susceptible peers–when conditions are favorable.

### Differential susceptibility in children with perinatal adversities

In a small-scale experimental study [5] mild perinatal adversities were defined as “children who were small for gestational age at birth and/or were born late preterm (born between the 34^th^ and 38^th^ week of pregnancy)”. Kindergartners (*N* = 100) both with and without perinatal adversities were subjected to a digital program stimulating letter knowledge and phonemic awareness (*Living Letters*). The effect of this intervention, as further described in [6, 7] was compared to the effect of a control program with digital storybooks (*Living Books)*, showing that children with perinatal adversities were differentially susceptible to the *Living Letter* intervention. In the control condition (*Living Books*), children performed significantly less well compared to their peers. In the experimental target condition (*Living Letters*) however, they significantly outperformed their peers, both in the short term (*Cohen’s d* = 1.24) and long term (one year after the intervention) (*Cohen’s d* = 1.11).

In a recent large-scale experimental replication RCT of [5], differential susceptibility of children with perinatal adversities to *Living Letters* was re-examined [7]. Participants with complete information in the study were 439 kindergartners, 142 of whom were children with perinatal adversities. Within the perinatal adversities group, 49 were children born late preterm and 102 were children born small for gestational age. Contrary to [5], results did not reveal differential susceptibility for the combined perinatal adversities group, nor for the children only born small for gestational age. However, differential susceptibility was found for the children born late preterm. Although the effect sizes in the replication study were substantial (*Cohen’s d* = .38 in the short term, and *Cohen’s d* = .37 in the long term), they were considerably more modest than those found in [5].

The discrepancies [5, 7] studies might be ascribed to differences in study design. For example, in [5], researchers supervised the implementation of the intervention, ensuring that digital sessions took place twice a week. In the replication study, as a practical consequence of the large sample size, teachers scheduled interventions sessions, which resulted in a less consistent dispersion across time, which in turn might have resulted in lower learning gains and thus reduced effect sizes. Additionally, in [5], posttests were administered by the researchers, whereas in the [7], posttests were administered by the teachers. Finally, [5], the posttests consisted of a large number of items (*k* = 40), while in [7], the posttests consisted of a much smaller number of items (*k* = 23). Fewer assessment items are generally associated with lower reliabilities, higher bias in scores, and less differentiation in skill levels [8], under the assumption that all items are of equivalent quality. The administration of posttests by the teachers rather than the researchers, and the smaller number of posttest items might have resulted in more noise in [7] replication study and might have influenced the magnitude of effects.

#### Planned missing data approach with gold-standard measures

One way to address the potential limitations of [7] was to use a planned missing data approach incorporating partially administered gold-standard measures. Planned missingness is a method to improve validity of results while maintaining the large power associated with larger sample sizes [9]. A planned missing approach with gold-standard measures involves the administration of an additional set of high-quality, ‘gold-standard,’ measures to a randomly selected subgroup of participants [10]. ‘Gold standard measures’ typically more expensive and time consuming to collect than other measures, but are also likely to provide more sensitive and valid information on the construct of interest. In a planned missing data approach, selection of participants who are administered the gold-standard measures is determined by the researchers completely at random and prior to study onset. Planned missingness, thus, relies on the presumption that gold-standard measurement data meet the criteria of being missing completely at random (MCAR), and hence that missingness is not associated with any bias [11]. Using scores from the less expensive (but possibly biased) measures as an auxiliary to the scores from the reliable, non-biased, gold standard, measures, a shared variance factor between the measures can be identified [10]. Graham et al. [12] argued that the two-method design relies on a gold-standard measure that is unbiased, supporting and correcting for other measure(s) that potentially contain systematic error. To gain full effect the central construct (e.g., literacy) and the bias are modeled as latent variables in a structural equation model, allowing to split the construct-related and non-construct-related variance of the inexpensive measures, yielding unbiased parameter estimates. Thus, the main gain of including such a gold standard measure is that it is expected to produce more accurate estimates of associations compared tonu the inexpensive measures [11], and, consequentially, the most accurate descriptions of individual effects.

Complete case analysis (CCA) commonly results in (strong) loss of power and an increased risk of biased results [13]. Therefore, actively addressing missing data tends to be preferred. Multiple Imputation (MI) [14] was long seen as inefficient in the current context, since it requires an imputation model in a complex multilevel setting, including interaction terms, which is not straightforward to set up. However, recent advances have provided new possibilities for multiple imputation using chained equations of fully conditional specifications [15, 16] for single level missing data as well as for multilevel and SEM models [17], even when such models include interaction terms [18, 19]. Pooling rules which closely adhere to Rubin’s rules are also implemented in software suites such as *lavaan* (version 0.6–7) [20]. Alternatively, using Full Information Maximum Likelihood (FIML), missing values are not replaced or imputed several times under a stochastic process, but missingness is handled within the analysis model [21], although some exceptions exist [22]. The FIML approach yields the most likely parameter values given all available data in the model, regardless of their level of completeness. In common linear models, MI and FIML will come to similar parameters estimates when outcome data is missing [23] and when the same information is incorporated in a multiple imputation model as in a full information maximum likelihood estimation [24]. Enders, Du & Keller [25] have shown that under multilevel structures and when using interaction terms, multiple imputation yields less biased results compared to maximum likelihood estimations.

### Current study

In the current study, we use a planned missing data approach with gold-standard measures to reexamine the data collected in [7]. For a randomly selected subsample of children from [7], trained research assistants administered an additional set of gold-standard early literacy measures in the areas of letter knowledge, phonemic awareness, and writing. We hypothesize that a missing data approach with gold-standard measures would offer a clearer and less biased pattern of effects.

Three research questions were addressed in the study:

1. Can the interactions between intervention (i.e. *Living Letters*) and the susceptibility factor (i.e. late preterm birth), as found in the Merkelbach et al. [7] replication study, be replicated utilizing a planned missing data approach?
2. Can an additional partial Gold Standard reveal interactions between *Living Letters* and other mild perinatal adversities, specifically being small for gestational age or being born late preterm?
3. Does addition of the Gold Standard augment the effect sizes from the large-scale replication design to those found in the original small-scale study?

## Methods

In this section, we describe the data collection methods used in the larger study [7], and additionally include the information specific to the current study, that is, to the gold-standard replication study. The current study was approved by the ethics committee of the Institute of Child and Education Studies from Leiden University.

### Participants

The sample from [7] study consisted of 981 five-year-old children. Subjects were excluded (n = 286) from the analysis due to non-consent for using their perinatal data. No significant differences (*p’s between* 0.083 and 0.674) between consenting and non-consenting participants were found on any of the model variables (see below for details on these variables). The remaining 695 participants included 82 children for whom no pretest scores were available, 45 children for whom no posttest scores were available, and another 129 children for whom consent was available, but perinatal information was incomplete. Participants were included in the main analyses after multilevel multiple imputation of the missing pretest, post test and perinatal data. Detailed are provided below.

Gold Standard results are available for a random selection of 443 out of 981 participants, in which the random selection was independent of availability of perinatal information. From these 443 participants with gold standard scores n = 201 are part of the subsample of participants with available perinatal information. The final sample, for which multiply imputed data on the predictive variables and the immediate post-test were available, consisted of 695 children from 180 different schools (% boys; mean age: months (*SD =*)). On average, there were 1 to 2 children per classroom in the study (*Mean* = 1.66 children per classroom, *SD* = .89). Gold standard measures were administered to a randomly selected (32.6%, *n* = 143) subsample of children (57.3% boys; mean age: 66.42 months (*SD* = 3.88)). Children in the subsample were from 54 different schools.

Sample characteristics of the complete-case participants in the available cohort (N = 981), the participants in the current analyses (N = 695) are presented in Table 1. Characteristics in the original analysis (*N* = 439) are equal to those reported in [7]. No differences between the experimental and control group were found. In subsequent analyses the multiply imputed multilevel data (N = 695) were used.

**Table 1:** Sample characteristics for the subsample with complete data, and compared per condition.

	Total cohort (N = 981)	Included group (N = 695)	Complete case group (*N* = 439)	Experimental: *Living Letters* (*n* = 230)	Control: *Living Books* (*n* = 209)	*p*
Male	55.2%	55.1%	55.4%	53.9%	56.9%	.524
Age (in months)	67.11 (*4*.*44*)	67.02 (*4*.*42*)	66.81 (*4*.*23*)	59.53 (*7*.*80*)	66.86 (*4*.*30*)	.793
Father’s education (max = 6)	3.78 (*1*.*67*)	3.76 (*1*.*67*)	3.71 (*1*.*38*)	3.74 (*1*.*42*)	3.69 (*1*.*35*)	.721
Late preterm birth	12.54%	26.04%	12.15%	12.6%	12.4%	.958
Small for gestational age	23.65%	35.12%	23.23%	22.6%	23.9%	.745
CLT pretest (raw score)	59.74 (*7*.*36*)	59.93 (*7*.*78*)	59.85 (*8*.*06*)	59.53 (*7*.*80*)	60.22 (*8*.*35*)	.372
CLT pretest (percentage low)	49.61%	49.74%	49.69%	50.38%	48.82%	.733
Alphabetic knowledge posttest (z-score)	N.A.	.00 (*1*.*00*)	.02 (*1*.*00*)	-.03 (*1*.*00*)	.02 (*1*.*00*)	.389

*P-*values indicate significance of the experimental versus control group differences. The sfga and preterm percentages in the included group are higher due to nonconsent participants dropping out after randomization.

From this group of participants in wave 2, we randomly selected a subsample consisting of 144 children (32.8%). In this subsample gold standard testing was administered. Sample characteristics of this gold standard subsample are reported in Table 2, and are, as is expected when missingness is at random, comparable to characteristic found in the complete sample. Within the gold standard sample, no differences between conditions regarding background characteristics (e.g. educational level of the father and age of the child) were found. However, on two of the three gold standard measures (i.e. word knowledge and word recognition) children in the *Living Books* (i.e. control) condition had higher scores than children in the *Living Letters* condition. This might suggest that in general, *Living Books* might have been better in stimulating these skills.

**Table 2:** Sample characteristics in the gold standard subsample, for the complete sample, and compared per condition.

	Complete group (*N* = 144)	Experimental: *Living Letters* (*n* = 75)	Control: *Living Books* (*n* = 69)	*p*
Male	56.9%	54.7%	59.4%	.565
Age (in months)	66.49 (*3*.*97*)	66.67 (*4*.*27*)	66.31 (*3*.*64*)	.549
Father’s education (max = 6)	3.76 (*1*.*38*)	3.80 (*1*.*43*)	3.71 (*1*.*32*)	.706
Late preterm	9.7%	10.7%	8.7%	.690
Small for gestational age	20.8%	18.7%	23.2%	.504
CLT pretest (raw score)	59.78 (*6*.*42*)	59.20 (*6*.*09*)	60.42 (*6*.*74*)	.256
CLT pretest (percentage low)	48.6%	54.7%	42.0%	.130
Alphabetic knowledge posttest (z-score)	.05 (.*93*)	.05 (.*93*)	.04 (.*94*)	.946
Word knowledge (gold standard)	.68 (.*12*)	.66 (.*12*)	.71 (.*12*)	.024
Word recognition (gold standard)	2.28 (.*51*)	2.17 (.*51*)	2.39 (.*50*)	.009
Writing (gold standard)	4.11 (.*88*)	4.05 (.*91*)	4.17 (.*85*)	.354

*p-*values indicate significance of the experimental versus control group differences.

No individual variables within the complete MPA subsample had deviating proportions of random missing values. Little’s MCAR test [26] shows that no patterns in missingness can be detected; χ^2^(27, N = 439) = 37.84, p = 0.08.

### Experimental design

In the current large-scale experimental design, children were randomly assigned to either the experimental condition (i.e. *Living Letters*) or the control condition (i.e. *Living Books*). For the larger study, teachers coordinated sessions and administered post-testing. Teachers were not informed about which program was considered to be the target condition or control condition, but were aware of condition to which children were assigned. The research assistants who administered the gold standard measures for the selected subsample of children were blind to the condition to which the child had been assigned.

### Procedure

**Fig 1:**
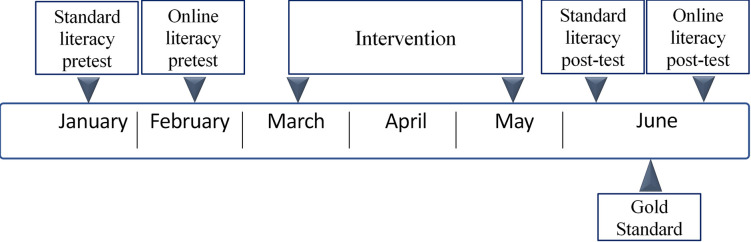
Study timeline. Timeline of the RCT replication study.

Data collection took place in two consecutive school years (2012/2013 and 2013/2014). The timeline is summarized in Fig 1. From August to February, schools were recruited by sending out flyers and letters containing information about the content and purpose of the study through both email and mail. Participating schools were offered three months of free access to all intervention programs, which normally require a paid subscription (http://www.bereslim.nl). When teachers agreed to participate, they were asked to select pupils from their classroom with poor language/literacy skills, for instance pupils who were not yet able to write their proper name, to rhyme, to name a few letters, and to identify sounds in words. Teachers were told that it was preferable that these children scored below the 40^th^ percentile (between 0 and 59) on a standardized Cito language/literacy test (CLT) that was administered in January in the schools [27]. If an insufficient number of children scored below the 40^th^ percentile, teachers were asked to include other children who they believed needed additional help with early literacy skills. Parents provided written informed consent for the child’s participation in the study. In Year 1, near the end of the study, parents also were asked for consent for retrieving perinatal information. Only 43% of parents provided consent for receiving perinatal information–perhaps due to the fact that the request was made at the end of the study. In the second year, parental consent for the child’s participation and for retrieving perinatal information both were requested at the beginning of the study. The vast majority of parents (94%) provided consent for retrieving perinatal information in the second year of the study.

Similar to [5], the current study contrasted *Living Letters* with *Living Books*. Other conditions included in the larger study are beyond the scope of the current work presented. Children were randomly assigned to a condition by the researchers. The intervention sessions took place once a week, and were spread over a period of approximately eight to twelve weeks. Except for logging in, children worked on their own without adult assistance. During the sessions, children wore headphones to prevent being disturbed by other children. Children worked with the mouse and did not have to make use of the keyboard.

At the end of the intervention period, teachers administered three digital tests measuring alphabetic knowledge and phonemic awareness (i.e. phonological skills, word recognition, and decoding) to participating children on an individual basis. Testing took approximately ten minutes for each child. Teachers were not allowed to help children; they were asked to only mark the child’s responses as either correct or incorrect.

Additional data from (a battery of) gold standard measures were obtained in a randomly selected subsample of children. In the full cohort just over 40% of children were randomly selected and received additional testing. After selecting only those children meeting criteria to answer the raised research questions–e.g. those assigned to the right conditions (*n* = 439), this percentage was around 33%. These additional tests were administered by highly trained research assistants. The gold-standard measures were three early literacy tests that targeted alphabetic knowledge and phonemic awareness. These measures are described in more detail below.

### Intervention programs

The target program, *Living Letters*, was designed to promote knowledge of the alphabetic principle and phonemic awareness in kindergartners. Two main characters, a boy and a girl, explain the assignments and an online tutor, the boy’s teddy bear, provides adaptive feedback after each assignment. Feedback is also given when the assignment is completed correctly. After the child provides the correct response, or the correct response is modeled, the teddy bear confirms that the answer is correct and explains why. If children provide incorrect responses in the games, the online tutor (the teddy bear) immediately provides feedback. In case of an incorrect response, three levels of feedback are provided: (1) first, repeating instructions; (2) second, providing cues to answer the question; (3) third, modeling the correct response. Feedback is provided in all games of *Living Letters*. In the first 22 games of *Living Letters*, children practice recognizing their own written name (or ‘mamma’) among other symbol strings or scribbles. The subsequent six games focus on the sound of the first letter of the child’s name. In the last twelve games, children select pictures of words that start or end with the first letter of their own name.

Control children received *Living Books* during the same period of time. *Living Books* includes eight digital, animated, age-appropriate stories based on high-quality children’s books. Each story is ‘read’ twice to the child via a computerized voice while children watch animations and listen to background sounds and music that support comprehension of the story content. The text is not presented as print on screen but only orally. Each reading session is interrupted four times so that children can answer two questions about the story events and two about difficult words in the text. After answering each question, children received immediate feedback, as well as positive feedback through compliments regardless of their individual performance.

### Measures

#### Pretest

At pretest, the Cito Literacy Test for Kindergarten Pupils (CLT) was used. The CLT is a group-administered test applied in January/February in the schools. The test consists of 60 paper-pencil questions measuring a range of language and literacy skills: vocabulary, critical listening, rhyming, hearing the first or last word in a sentence, sound blending, writing conventions, and prediction of book content based on book cover [28]. Children’s pretest score was coded as scoring among the lowest 25% (score of 59 or below) or average (score of 59 and beyond). A dichotomous score was used to adhere to diagnostic information as used in practice (schools) as closely as possible. A full range score was also modelled, yielding equivalent outcomes.

#### Posttest: Entire sample

A battery of early literacy measures was administered by teachers to all participating children in the study. The battery included a phonemic awareness task, a letter knowledge task, and a word recognition task.

*Phonemic awareness*. The Phonemic Awareness Task included five items. Children identified the first sound of five words (e.g. muis [mouse]) while pictures of the words were shown on the computer screen. Cronbach’s *α* was .76.

*Letter knowledge*. Children identified ten letters presented on screen (i.e. *s*, *k*, *a*, *p*, *r*, *o*, *v*, *m*, *t*, *& n*). Cronbach’s *α* was .83.

*Word recognition*. Children were asked to match a printed word with picture. For each of six words (e.g. dak [roof]) there were four options (one correct, three incorrect) from which they could choose. The incorrect options varied in systematic way: no letter correct (lom), first letter correct (dor), first and last letter correct (dek). Cronbach’s *α* was .83.

*Aggregate measure*. Principal component analysis (PCA) applied to the three tests resulted in one component explaining 67.59% of the variance. Component loadings ranged from .74 to .86. Resulting component scores are standardized weighted composites, in which a higher score indicating better alphabetic skills.

#### Posttest: Gold standard measures for randomly selected subsample

In addition to the measures described above, three gold-standard measures were administered by research assistants to individuals in a randomly selected subsample. These three measures included a vocabulary, a word recognition and a writing measure.

*Vocabulary*. The vocabulary word knowledge test consisted of 25 items in which a sentence derived from a digitally animated storybook, was read to the child, after which a target word was repeated, and children were asked to give a definition of the word (e.g. ‘Are you lost little one?’ the bear asked kindly. What does *lost* mean?’). Answers were scored as correct (1), partly correct (.5), or incorrect (0). *Cronbach’s α* was .72. For no item did deleting the item result in a higher Cronbach’s *α*.

*Word recognition*. Ten word-recognition items were administered to the students for the gold-standard word recognition test, including the six items used in the teacher administered test and four new items. As with the task administered by the teachers, children were asked to match a printed word with a picture. For each word there were four options, and the incorrect options varied systematically. Cronbach’s *α* was .74.

*Writing*. The writing test, developed by [29], consisted of six items asking children to write their own names and five other short words. Items were scored on a seven-point scale with a score of 0 indicated drawing and a score of 6 indicating a completely correct written word. Cronbach’s *α* was .80).

*Aggregate measures*. A total of three planned missing data models were fitted. In the first model, a two-factor approach was used, in which the word knowledge task was considered as one factor (measuring vocabulary, *Cronbach’s α* = .73), and word recognition and writing were combined into another factor (targeting alphabetic knowledge and phonemic awareness, *Cronbach’s α* = .79). In the second model, only the factor measuring alphabetic knowledge and phonemic awareness was used. In the third model, only the writing score (*Cronbach’s α* = .83) was used because it most closely approached the skills trained by *Living Letters*.

#### Mild perinatal adversity

Conforming to prior definitions [5, 7], mild perinatal adversities were defined as children who were small for gestational age (sfga) at birth and/or were born late preterm (preterm: born between the 34th and 38th week of pregnancy).

### Statistical analyses

#### Preprocessing and main analyses

As in preceding studies, to test effects of *Living Letters*, a multilevel approach using mixed models was applied to account for variance attributable to school-level characteristics [30]. We employed a likelihood ratio test to examine model improvement when intercepts or intercepts and slopes were allowed to vary across schools. The following variables were included in the analyses: pretest score, condition, sfga and preterm classifications, and two two-way interactions (small for gestational age*condition, late preterm*condition). Separate analyses were performed for sfga and preterm separately to assess robustness of the main results. Analyses were repeated for DRD4 as a moderator to assess specificity.

Incomplete data for pretest, posttest, perinatal information and their interactions were imputed in the consenting participants using BLIMP 2.2, all accounting for the multilevel structure due to nesting within schools. [25] describe the benefit of using model-based imputations to reduce bias in imputation of interaction terms. As our analyses contain multiple models, each including a different but specific interaction, these cannot be captured in a single specification for imputation. This would lead to model and result comparison based on different imputation sets, which is suboptimal. The alternative, using multiply FCS imputed data with prespecified interaction terms, might yield (slightly) biased imputations, also being suboptimal. Therefore, we compared estimations from both aproaches, which yielded equivalent results. To match day-to-day use–in which several questions would be answered from a single dataset—we ran our final analyses on the FCS imputed data, to ensure that all random variability across imputations was the same at all time. The FCS imputations were run in BLIMP using 5000 burn-in runs for 4 chains in the MCMC Gibbs sampler, followed by 100 imputation iterations. The PSR and diagnostic plots showed stabilization after around 3500 burn in iterations. A total of 100 sets was generated into separated files. Imputation set files were read into R separately and were internally combined into a list of sets, in order to achieve compliance to the internal pooling requirements.

We adopted FIML for the gold standard inclusion to include the Gold Standard measures in the pooled model results.

#### Gold standard models

For model estimations, we used *lavaan* 6.70 in R version 4.02. Full information maximum likelihood was used to use all participants account for missing data in the Gold Standard definitions. The number of EM iterations was set to a maximum of 5000. All models were fitted on all 100 imputed datasets and their results were pooled using the *.*mi* functions from the *semTools* package, with the D3 estimator.

**Fig 2:**
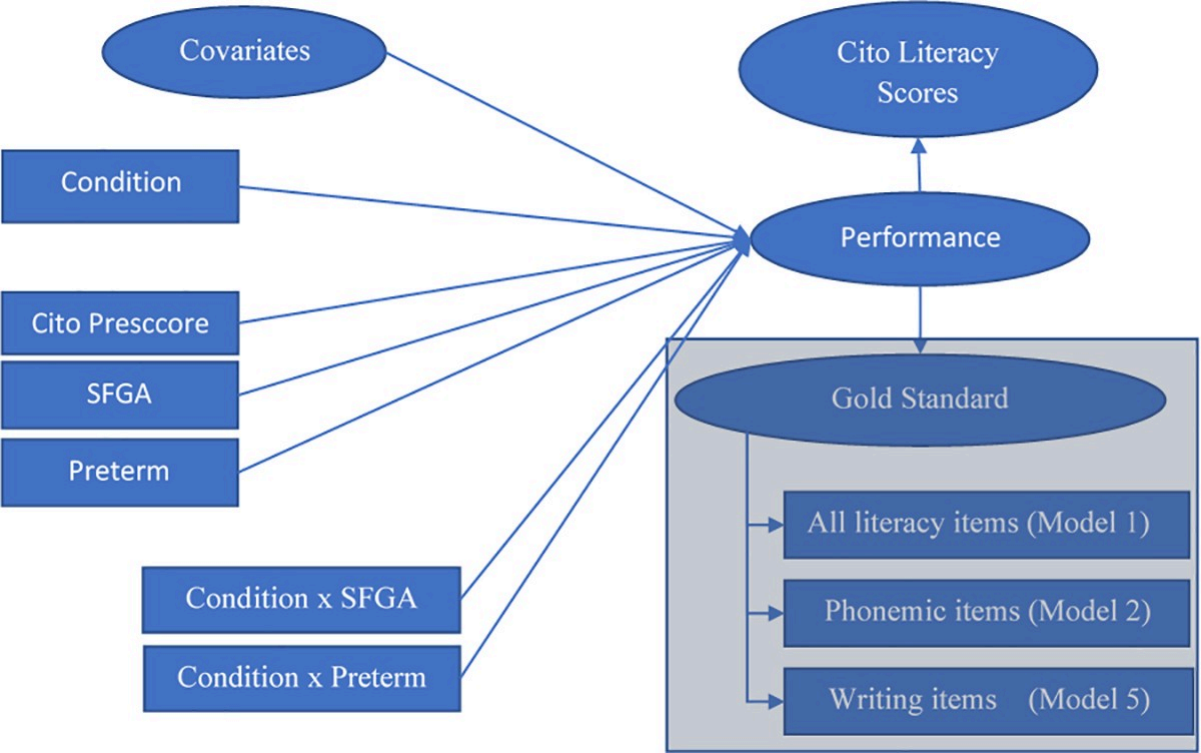
Model diagram. Baseline model. The bottom right block-filled area indicates the extension of the baseline model with two of the main definitions of Gold Standard scores.

To evaluate replicability when using the gold standard, five model variants were fitted to the data, depicted in Fig 2. All models accounted for nesting within schools and were adjusted for differences in age and gender. In the first model (1) a very general composition of the gold standard, using all measurements administered during the gold standard test sessions, was considered, thus exploring whether adding broad gold standard literacy measurements, not directly related to the intervention per se, could improve the model fit. Test scores were split into two factors describing different components of early literacy development (i.e. one factor focussing on vocabulary (Cronbach’s *α* = .78), and another factor focussing on word recognition and writing (Cronbach’s *α* = .89), which are both skills relying on alphabetic knowledge and phonemic awareness). In the second model (2) the two separated factors were combined into a single factor score for the Gold Standard by forcing a 1-factor solution. In the third model (3) the second factor from model 2 was split to represent only word picture recognition in combination with Writing skills. In the fourth model (4) the second factor from model 2 was split to represent only word range in combination with Writing skills, and in the final model (5) the gold standard factor consisted of Writing only, because this measure differs from the assessment by the teachers and is known to be a strong indicator of alphabetic knowledge.

#### Model evaluation

The fit of the baseline model without gold standard data (0), hereafter ‘baseline model’, and the fit of the five gold standard models were compared using classical evaluation criteria and the equivalence tests for CFI and RMSEA [31, 32]. To determine model performance we assesed the relative fit of each model through the Comparative Fit Index (CFI) and its robust equivalence counterpart (t_CFI).The absolute fit was assessed using the Root Mean Square Error of Approximation (RMSEA) and it’s robust equivalance counterpart (t_RMSEA). The classical CFI should be as high as possible (ideally above .90), while the sRMR and the classical RMSEA should be as low as possible (ideally below .06). The cutoff values for the t_CFI and t_RMSEA in the current sample and model definitions are provided in Table 3.

**Table 3:** Cutoff values and interpretation for CFA and RMSEA equivalence test.

	*99.9% CI*	*99% CI*	*95% CI*	*90% CI*
*interpretation*	excellent fit	close fit	fair fit	mediocre fit
*t_CFI*	0.968	0.909	0.867	0.840
*t_RMSEA*	0.036	0.068	0.097	0.116

Values are specific for the current sample and models.

*Evaluating efficiency and bias*. Relative efficiency is typically a ratio of standard errors from two different estimators, referring to the precision with which parameters are estimated and is related to the information available in the data. Thus, efficiency is a centrally important concept whenever planned missing data designs are evaluated. Adding an unbiased measure is expected to improve parameter estimates compared to models with only the biased measures. Adding more participants with an inexpensive measure is expected to improve efficiency, relative to a smaller sample with just the gold standard measure. Model fit is not expected to be systematically affected, in fact, all these models would be expected to have similarly good fit. Model fit does not improve due to estimates getting more precise or stronger, or due to a decrease in residual variance.

*Comparing results*. Once determined if and how planned missing data models were able to improve the fit of the baseline model (0), and selecting the best fitting gold standard model, individual parameters obtained from the baseline model (0) and from the best fitting model(s) were compared to determine whether results could be replicated using a gold standard approach.

## Results

### Model fit

Model fit results for the model estimations including both mild perinatal adversities are summarized in Table 4. The five gold standard models, as well as the original baseline model (0), show reasonable to (very) strong fit according to the classical CFI criteria. However, according to the equivalence tests models 0, 2 and 5 show a close fit (CFI>0.91) and model 5 shows excellent fit (CFI>0.97). According to the classical RMSEA all models except model 3 show a good fit. Using equivalence tests all models except model 3 show a close fit, and none of the models show an excellent fit. Models 2 and 5 show equivalent AICs, while model 5 show higher t_CFI (0.97 vs 0.94) and lower t_RMSEA (0.043 vs 0.065) compared to model 2.

**Table 4:** Model fit statistics: Comparing fit of baseline model (0)—without gold standard, and gold standard models 1, 2, 3, 4 and 5.

Model	*CFI*	*T_CFI*	*RMSEA*	*T_RMSEA*	*AIC*
Model 0: Baseline	0.97	0.91	0.042	0.060	3514.75
Model 1; 2 factors	0.98	0.74	0.046	0.077	1427.24
Model 2; 1 combined factor	1.00	0.94	0.000	0.065	1249.09
Model 3; word recognition & writing	0.96	0.76	0.066	0.094	1931.09
Model 4; word range & writing	0.97	0.69	0.049	0.080	1678.58
Model 5; writing	1.00	0.97	0.000	0.043	1249.86

We consider the fit of the baseline model (0), as well as the fit of gold standard model 2 (one factor including word recognition and writing), and gold standard model 5 (writing only), as satisfactory. We can conclude that both gold standard model 2 and gold standard model 5 are significant improvements over the baseline model (0), with model 5 showing the best relative performance and fit.

Results for moderator SFGA and Preterm separately, as well as for DRD4, are provided in S1 File.

### Efficiency

As can be seen in Table 5, the standard error goes down from model 2 to 5 for all estimates except the CITO pretest parameter. Thus model 5, including the highly specific gold standard writing scores, is the model to be favoured over others to describe the differential performance patterns for early literacy intervention.

**Table 5:** Comparing pooled results of analysis in the baseline model (0)—without gold standard, and gold standard models 2 and 5.

	Baseline Model (0)		Model 2 (1 factor)		Model 5 (writing)	
	Est (*SE*)	*p*-value	Est (*SE*)	*p*-value	Est (*SE*)	*p*-value
CITO Pretest	.58 (.11)	< .001	.58 (.11)	< .001	.57 (.16)	< .001
Preterm	-.04 (.11)	.689	-.01 (.19)	.964	.01 (.18)	.974
SFGA	-.07 (.06)	.520	-.10 (.17)	.525	-.17 (.15)	.239
Condition	-.01 (.05)	.922	.01 (.12)	.941	-.04 (.12)	.705
Preterm * condition	.07 (.05)	.154	.13 (.07)	.053	.14 (.06)	.019
SFGA * condition	.01 (.05)	.833	.05 (.08)	.561	.03 (.07)	.710

### Replication of prior study results

As model 2 and 5 showed comparable fit with the baseline model (0), and showed an increase in relative efficiency as compared to the baseline model (0), only results of these models are compared to the baseline model (0) (Table 5).

Pooled estimates (and standard errors), presented in Table 5, are highly comparable across all three models, showing that in general the analysis yielded similar results. In all models, pre-test was a significant predictor. However, the interaction between late preterm and condition (*Living Letters* vs. *Control program*), which was not significant in the baseline model (0) (*p =* .154) and just not significant in gold standard model 2 (*p =* 0.053), was however found to be significant in model 5 (*p =* .019), conditional for all other effects in the model. The interaction between small for gestational age and condition was not significant in any of the models, including the most accurate representation in model 5 (*p* = 0.561).

## Discussion

The aim of the current study was to examine whether results of a large-scale intervention would be replicated, specifically whether effects of the intervention would be moderated by child characteristics. Specifically, we tested whether we could replicate the interaction effect between *Living Letters*, a digital intervention program promoting alphabetic knowledge and phonemic awareness, and late preterm birth, using a planned missing data approach. Results were replicated, however not in all planned missing data models fitted to the data.

To evaluate potential replication, three planned missing data models, differing in the amount and accuracy of gold standard data included, were fitted to the data. All models showed reasonable to very good absolute fit, while the fit improved with increasing specificity of the gold standard measure. Only in two of the fitted models the relative efficiency of the model improved when compared to the baseline model (0). In one of the planned missing data models–the model in which the broadest range of gold standard data was included–model fit was insufficient. This implies that the gold standard data did not approximate the skill-set stimulated by *Living Letters* (i.e. word knowledge). These findings demonstrate that obtaining more, but possibly less relevant, information does not always lead to model improvement, and thus that selection of tests to serve as gold standard measurements should take place with caution. Because gold standard data are assumed to be measured without bias [11], high quantities of information with limited validity are not preferable to using less information with higher levels of construct validity. In the two models that showed improvement of model efficiency, estimates and thus effect sizes were comparable to those in the baseline model (0) (described in [7]. The results from the model with a general gold standard are based on the same subsample as the one used in the model with the “writing-only” gold standard. In the first model there are no sign flips compared to the model without gold standard. This can be considered an argument against direct risk of a biased subsample. The specific patterns found in the writing-only gold standard model sometimes differ in direction compared to those in the model using the general factor. In the latter model the specific effects of writing are outweighed by the extended information in the factor. This internal factor structure is hence more noisy than the writing factor, which would also explain the better fit for the model with a specific gold standard over the model with a general gold standard. The main finding, a significant interaction between condition and late preterm birth, was found in all planned missing data models, but was most efficient in the model including only the measurement with the highest level of convergent validity–writing.

Additionally, we explored whether using a planned missing data design would reveal interactions between *Living Letters* and being small for gestational age. However, as in the analysis without missing data, in all three planned missing data models, this interaction remained non-significant. Because p-values are very large, further improvement of power is not expected to result in a detectable manifestation of this interaction.

Lastly, we tested whether effect sizes would approach effects found in [5] if a planned missing data approach was used to improve design validity. We would expect clearer effects if bias, and thus measurement error, might possibly explain the reduced effect sizes [33] of the Merkelbach et al. replication study [7] when compared to the original experiment [5]. However, using a planned missing data approach did not result in the emergence of clearer effects. We might thus conclude that bias and measurement error cannot explain the discrepancy between the two original studies. This suggests that neither the way teachers administered tests, nor the validity of the original posttests, were factors that likely influenced the results. It is possible that the discrepancies in the results between these two studies might thus be explained by other factors, such as the quality of implementation of the intervention (i.e. less consistent dispersion of sessions when teachers coordinate the intervention).

## Conclusion

In the current study we tested whether, using a planned missing data approach, we would replicate results of a large scale RCT examining the differential effects of a digital early literacy intervention focused on alphabetic skills and phonemic awareness. Three planned missing data models were fitted to the data of the large-scale RCT. Model fit did improve by including a gold standard, through which results could also be replicated. However, adding a gold standard did not result in effect sizes matching those found in a previous small-scale, potentially underpowered, study, suggesting that bias and measurement error did not account for the differences in effect sizes found between the original and the replication study.

In the most efficient model in which replication was found, only gold standard data with high convergent validity were included (i.e. writing), while gold standard measures approaching the skill trained by the intervention less closely (i.e. word knowledge) were not included. Planned missing data approaches in replicating RCT-studies can thus be useful, but only when used with care: Previous findings might be replicated using a planned missing data approach, however, only when only gold standard testing closely approximating the trained skills at hand are included.

## Supporting information

S1 File Model for gold standard models for SFGA, preterm and DRD4 as separate moderators. (PDF)

S2 File Dataset with selected variables. (CSV)
