# Supplementary material for: Planned missing data in early literacy interventions: A replication study with an additional gold standard
Source: PLoS One. 2021 Mar 29;16(3):e0249175. doi: 10.1371/journal.pone.0249175 (PMC8007029; doi:10.1371/journal.pone.0249175)
Supplement: S1 File — (PDF) [file pone.0249175.s001.pdf]

*S1 File: Model comparison using subtypes of MPA data and DRD4 data*

**Planned missing data in early literacy interventions: A replication study with an additional gold standard - Ralph C. A. Rippe & Inge Merkelbach**

**For robustness**

| **Models for SFGA** | *CFI* | *T_CFI* | *RMSEA* | *T_RMSEA* | *AIC* |
|---|---|---|---|---|---|
| Model 0 | 0.97 | 0.93 | 0.056 | 0.077 | 3495.22 |
| Model 1 | 0.98 | 0.89 | 0.051 | 0.086 | 1471.36 |
| Model 2 | 0.99 | 0.95 | 0.025 | 0.078 | 1239.21 |
| Model 3 | 0.96 | 0.86 | 0.082 | 0.113 | 1921.20 |
| Model 4 | 0.97 | 0.88 | 0.053 | 0.088 | 1668.69 |
| Model 5 | 1.00 | 0.85 | 0.000 | 0.088 | 1376.34 |

| **Models for Preterm** | *CFI* | *T_CFI* | *RMSEA* | *T_RMSEA* | *AIC* |
|---|---|---|---|---|---|
| Model 0 | 0.97 | 0.93 | 0.055 | 0.076 | 3492.27 |
| Model 1 | 0.97 | 0.87 | 0.064 | 0.096 | 1417.62 |
| Model 2 | 0.99 | 0.95 | 0.031 | 0.081 | 1239.48 |
| Model 3 | 0.96 | 0.86 | 0.086 | 0.116 | 1921.48 |
| Model 4 | 0.97 | 0.86 | 0.068 | 0.099 | 1688.93 |
| Model 5 | 1.00 | 0.84 | 0.000 | 0.093 | 1377.44 |

**For Specificity**

| **Models for DRD4** | *CFI* | *T_CFI* | *RMSEA* | *T_RMSEA* | *AIC* |
|---|---|---|---|---|---|
| Model 0 | 0.97 | 0.94 | 0.065 | 0.087 | 3475.83 |
| Model 1 | 0.98 | 0.91 | 0.066 | 0.101 | 1412.43 |
| Model 2 | 1.00 | 0.97 | 0.000 | 0.069 | 1234.27 |
| Model 3 | 0.96 | 0.89 | 0.088 | 0.121 | 1916.29 |
| Model 4 | 0.97 | 0.90 | 0.069 | 0.104 | 1663.74 |
| Model 5 | 1.00 | 0.98 | 0.000 | 0.059 | 1234.98 |
